# Clinicians’ Perspectives on Self-Harm in Pakistan: A Qualitative Study

**DOI:** 10.3389/fpsyt.2021.607549

**Published:** 2021-05-20

**Authors:** Tayyeba Kiran, Nasim Chaudhry, Penny Bee, Sehrish Tofique, Sana Farooque, Afshan Qureshi, Anna K. Taylor, Nusrat Husain, Carolyn A. Chew-Graham

**Affiliations:** ^1^Pakistan Institute of Living and Learning, Karachi, Pakistan; ^2^Division of Nursing, Midwifery and Social Work, University of Manchester, Manchester, United Kingdom; ^3^Division of Psychology and Mental Health, University of Manchester, Manchester, United Kingdom; ^4^School of Medicine, Keele University, Keele, United Kingdom

**Keywords:** self-harm, Pakistan, clinicians, lower middle income country, qualitative methods (interviewing)

## Abstract

**Background:** Suicide is a serious public health problem, ranked amongst the leading causes of death worldwide. There are no official data on self-harm and suicide in Pakistan; both are illegal acts, and are socially and religiously condemned. This study explored the views of clinicians, including general practitioners (GPs) and hospital physicians (HPs) on self-harm, about their management of people who self-harm and what interventions might be appropriate in Pakistan.

**Methods:** This qualitative study, generating data using semi-structured interviews, was nested within a Randomized Controlled Trial (RCT) of a psychosocial intervention for people following self-harm. Clinicians (*n* = 18) with experience of treating people who self-harm were recruited from public hospitals and general practices.

**Results:** Face-to-face interviews were conducted in Urdu and digitally recorded with consent, transcribed and translated into English. Transcripts were checked for cultural and interpretive interpretations by the research team, then analyzed thematically using the principles of constant comparison. The following themes will be presented: encountering people with self-harming behaviors; challenges encountered in managing people who self-harm; barriers to accessing care, and what ideal care might look like. Participants identified their lack of training and expertise in the management of people with self-harm behavior.

**Conclusions:** This is the first study to explore clinicians’ perspectives on self-harm in Pakistan. The study highlighted the need for training for doctors in the identification and management of mental health problems, including the management of people who self-harm.

## Introduction

Suicide and self-harm are serious global public health concerns. The World Health Organization’s Suicide Report estimates that more than 800,000 people kill themselves annually worldwide, with the majority of these cases (75%) in low and middle income countries (LMICs) (1). Prior history of self-harm or attempted suicide are strong predictive factors of future death by suicide, increasing risk by up to 100 times (2, 3). Each suicide takes the life of the individual, and impacts on family, friends, healthcare providers and the wider community (4, 5). The WHO’s Mental Health Gap Action Programme includes suicide as one of its priority conditions, and the WHO cites suicide prevention as one of its key Sustainable Development Goals (SDGs) (6). There is accumulating evidence that suicide rates are increasing in Pakistan (7, 8).

In Pakistan, a conservative South Asian Islamic country, no official data is available on self-harm and suicide. There is stigma around mental illness in Pakistan, which creates individual barriers to help-seeking (9). According to the Pakistan Penal Code, both suicide and attempted suicide remain illegal acts. These acts are socially and religiously condemned. There is the concept of shame around reporting the act of self-harm because of the ingrained notion of izzat (honor) in Asian families, and Pakistani women in particular have very limited access to healthcare when compared with other South Asian countries (10, 11). Previous literature includes a qualitative study in which semi-structured interviews with Indian Gujarati participants were conducted to explore their views on barriers and facilitators in accessing mental healthcare. Participants reported the concept of honor and reputation in South Asian communities as major barriers in accessing help (12). In a focus group discussion with South Asian women, participants expressed that fear of “being found out”; the power of izzat was a major barrier in accessing help for mental health problems (10).

Psychiatric disorders commonly go undiagnosed and untreated in Pakistan (13). Expertise in assessment and treatment of people with mental health problems is sparse (14, 15). Referral pathways to secondary psychiatric care exist, but are under-utilized because of low rates of case-finding (15, 16). The attitudes of clinicians toward people with episodes of self-harm, as well as their knowledge about self-harm, have important influences on their clinical practice and hence on the experiences and outcomes of those they treat. Existing evidence suggests that clinicians’ classification of risk of suicide was inaccurate (17, 18). There is no previous literature on the attitudes of clinicians toward people who self-harm in Pakistan, and this remains an important knowledge gap.

This paper reports a qualitative study nested within a multicentre randomized controlled trial of a culturally adapted manual assisted problem solving intervention (CMAP) for prevention of self-harm (trial registration number: NCT02742922). This study aimed to explore how hospital/general physicians and general practitioners (GP) in Pakistan respond to, and manage people with self-harm behavior.

## Materials and Methods

### Ethical Approval and Consent

Ethical approval for the study was granted from the Institutional Review Board of Karachi Medical and Dental College (KMDC) (Ref: 027/15), Karachi, Pakistan, and ethics committee of University of Manchester (UoM). Consent to participate and publish was gained from the participants.

### Design

A nested qualitative study within a Randomized Controlled Trial (RCT).

### Study Site and Population

Hospital physicians working in medical units of public hospitals participating in the CMAP trial, and GPs working in general practices (located in the vicinity of hospitals participating in the CMAP trial) were invited to participate in a single semi-structured interview. Convenience sampling was used, and participants were recruited depending on their availability and willingness to participate in an interview.

A Participant Information Leaflet was provided to all participants at the time of invitation and any queries were addressed by the researcher. Written consent was obtained to digitally-record the interviews. Semi-structured interviews were conducted by experienced researchers (qualified clinical psychologists, trained and experienced in qualitative data collection and analysis).

The interviews offered the opportunity to discuss, in-depth, a range of topics relevant to the research questions, but also allow for exploration and probing of issues raised during the interview. The topic guide was developed by the research team from the literature and through discussion. The topic guide covered hospital physicians and GPs’ understanding of the term “self-harm,” perceptions about reasons for self-harm, and exploration of current pathways to care and management of people with self-harm behaviors. The topic guide was modified and refined in the light of interim analysis and emerging themes. All interviews were conducted and transcribed verbatim in Urdu, translated into English (by TK and NC who are both fluent in English and Urdu), and then back-translated to check accuracy of translation, prior to data analysis. All interviews were conducted by researchers (ST, SF) in Urdu. Interviews were continued until data saturation was achieved (19, 20).

### Analysis

Data analysis following an inductive and interpretive thematic approach (21, 22), based on the principles of grounded theory (23, 24) making use of the research data to generate theory. However, the approach was not purely grounded theory, as we drew on the literature and our professional backgrounds in developing the topic guides for the study. The process of analysis was iterative as data collection progressed, using the principles of constant comparison (23), until data saturation was achieved (19, 20). We reached data saturation in the two separate data-sets (hospital clinicians and GPs) and initially analyzed the two data-sets separately. We then compared themes across the two data-sets in order to compare and contrast perspectives.

During the initial familiarization stage, three Urdu-speaking researchers (TK, ST, SF) read through the transcripts and field notes collected during the interviews several times to fully immerse themselves in the data. After researchers had familiarized themselves with the data, line-by-line coding was conducted. Initially, three transcripts from each data-set (hospital physicians and general practitioners) were coded independently by two coders (ST, SF), and discussed to ensure accuracy and consistency. The remaining transcripts were then divided between these two coders for individual coding. A draft framework was constructed in which major and minor themes were identified from all interviews. These data management tables outlined themes and supporting data, along with narrative explaining the meaning of the themes.

Indexing was then carried out in order to apply the draft theoretical framework systematically to the whole data-set. Here the data from transcripts were copied and pasted alongside the relevant themes that were listed in the draft framework. Data and themes were then compared again and the analysis framework revised. During the charting process, data were summarized into tables developed using MS Word software for each theme listed in the analysis framework.

This process provided a clear and concise overview of the data. Finally, tables were reviewed during the mapping and interpretation phase. This enabled all key ideas and the data to be compared and discussed within whole research team, and to identify the final framework that synthesized and interpreted the data as a whole.

To maintain credibility and trustworthiness of the data and subsequent findings, the researchers in Karachi were supervised by an experienced mental health professional (NC) and senior researchers with expertise in qualitative methods (CCG, PB) based in the United Kingdom. These researchers reviewed all transcripts, and a sample of the transcripts was discussed in regular Skype meetings. Engagement in discussion and regular reviews by all researchers ensured fit of the data to the final analysis, and helped to minimize bias (25). Research team members agreed key themes and interpretations.

## Results

Seven GPs and eleven hospital physicians were invited and interviewed between February and December 2017. All those invited agreed to participate in the interviews. Four GPs were male and three were females. Seven hospital physicians were female and 4 were male.

The average number of years the GPs had been in practice was 9.3 years (range 2 to 18 years) and hospital physicians 6.7 years (range 1 to 15 years). The mean duration of the interviews was 39.1 min.

The themes derived from the data are: encountering people with self-harm behaviors, including methods, views on the triggers of self-harm and beliefs about people who self-harm; challenges in managing people who self-harm; barriers to accessing care; and suggestions for an ideal care pathway.

Verbatim data extracts are presented to support the analysis, with participant identification numbers given.

### Encountering People With Self-Harm Behaviors

#### Methods of Self-Harm

Participants described a range of methods of self-harm in patients they had encountered including poisons (bleach, insecticides, organic phosphate compounds, blue stone, rat poison, kerosene, petrol, and copper sulfate) and overdoses of prescribed medication (benzodiazepines, aspirin, and medicines prescribed for physical long-term conditions). More violent methods described included hanging, gunshot, cutting, and jumping from a height, which were reported to be used more often by males.

“Gender difference is that mostly males don’t take drugs to attempt suicide, while females in the majority take sleeping pills, benzodiazepine, valium, phenyl if they have it at home. The majority of males attempt suicide by gunshot.” (GP007)

#### Triggers of Self-Harm

Different triggering factors were identified as making a person vulnerable to, or lead them toward self-harm. Factors described by some participants included severe mental health problems:

“It only happens when the patient is suffering from a severe mental health problem like schizophrenia.” (HP03)

“As a patient they are very disturbed psychologically and mentally that they don’t find any other option except taking their life. They are in a depressive phase.” (GP004)

Other participants suggested that people with self-harm behaviors, however, were motivated by the need to “seek attention” or express discordance with their family:

“Most of the time it’s just to make their family members scared, to seek attention or to convince someone.” (GP002)

A number of participants cited economic or financial difficulties as the immediate precipitant of an act of self-harm:

“Financial pressures related to increasing expenses both for males and females.” (HP008)

However, most participants suggested that self-harm was usually the result of a complex inter-play of multiple factors:

“Because of financial issues, unemployment, divorce, and children taken away from the wife due to divorce, adjustment at parent’s home after marriage when the girl comes back due to divorce and is teased about that and similar reasons. And in the younger generation problems like relationships, getting scolded by parents or fights with siblings or issues in education.” (GP003).

#### Beliefs About People Who Self-Harm

When asked about their views on why people self-harm, some participants expressed that self-harm was a consequence of poor problem-solving skills:

“Absolutely there is a strong relationship between problem solving abilities and self-harm. When they do not know how to solve problems then they only have the option to harm themselves.” (GP005)

Other participants suggested that self-harm was conducted as a means to threaten families:

“Do it basically to threaten their family because they take it in less quantity, otherwise if someone really would want to harm themselves then you wouldn’t be able to stop those patients.” (GP003)

Participants proposed that self-harm in females was much less well-tolerated by the family than self-harm in males, however, family also react when a male member attempts self-harm:

“People obviously don’t find it good. If it’s a girl then they assume that her character is not good and start gossiping of the reason why she attempted self-harm, like relationship problems or trauma.” (GP006)

“Family shows concern. In the case of females, family is concerned, and in the case of males, the family’s response is aggressive and they usually beat them.” (GP004)

### Challenges in Managing People With Self-Harm Behaviors

#### Self-Harm Is Hidden

Participants described self-harm as a hidden problem and suggested a variety of reasons for this. Because self-harm is illegal, participants suggested that families were reluctant to disclose self-harm in their family members as this would lead to involvement of the police.

“Especially in female cases and young girls and students, their families do not want to get in trouble with the police or court, which can create more trouble in their life. So they try to hide the cause and what actually happened with the victim and in the end they lose their life or have disability.” (GP002)

“People tend to hide a lot. They never tell you the exact thing or the situation. Specially, if their family members are present. They always say it was done by mistake. You only find this out when you probe the individual or their family a lot.” (HP010)

#### Managing an Illegal Act

A key challenge reported by participants was the problem that self-harm is an illegal act in Pakistan and private hospitals and clinics may avoid treating such patients.

“The problem that we face during treating patients with self-harm in private hospitals and clinics is that the government does not permit treatment there.” (GP005)

“Under the law it is required to have a legal medical certificate that is kept as a record of the patients that we work with. Apart from having a certificate to admit the patient, we also need to inform the police depending on the case we are dealing with.” (HP001)

#### Service Level Challenges

Because of the reported methods of self-harm, participants reported that it was challenging to manage these patients because of limited availability of space and lack of functional care in intensive care units in Pakistan:

“We have to admit patients in a critical situation to the intensive care unit, and at times all the beds are occupied. Because of such situations we have to refer patients somewhere else or the patient might expire.” (GP001)

For all patients encountered who have self-harmed, participants recognized that psychological input was necessary, but access to psychological services was reported to be limited, so hospital physicians and GPs reported that they had to provide psychological support, for which they had received no training.

“In small cities where I have worked there was no psychologist for providing psychological services and people need psychological support at that time, so we provide support to them as best we can.” (HP004)

### Barriers to Accessing Care

In addition to the fact that self-harm is an illegal act, thus posing a major barrier to accessing care, participants cited other reasons that prevented people who self-harm from help-seeking. Families usually do not accept self-harm as a psychological problem; rather they view this as attention seeking and hence they are in a state of denial where they are unable to provide support.

“Some families appear resistant; they don’t want even to listen and they deny the situation that has happened. Some families consider this act as malingering. They consider it as drama and they don’t show care for the person.” (GP001)

The attitude of the family and stigma related to self-harm, suicide and psychological services were frequently mentioned. They were particularly concerned about the community grapevine and fear of people finding out, which would have a negative effect on their family’s izzat (honor):

“Firstly, the stigma. People are afraid about what will happen if others come to know that they visited a psychiatry department because a family member has committed self-harm.” (GP006)

“There is a huge social stigma. Families are afraid that this will spread all over the village that their daughter tried to commit suicide. People will ask. People will talk.” (HP007)

The lack of awareness around role of psychiatrists was also raised as an issue contributing to social stigma as those seeing a psychiatrist are labeled as “mad” or “crazy.”

“With the psychiatrist there is a social stigma attached that those who go to them are crazy and this is something really difficult to make educated people understand as well. What is the definition of a psychiatrist?” (HP008)

Participants reported that money is essential for treatment such as to cover transport expenses to commute to the hospitals and the medication cost, and most people have insufficient resources to meet these expenses. Therefore, lack of money was also perceived to be a barrier to help-seeking:

“There are so many poor people we have seen that they find it difficult to reach hospital because of their financial limitations. They don’t have healthcare providers because they don’t have enough money for transport to reach the hospital.” (GP001)

Religious and spiritual beliefs were commonly mentioned as factors that deterred patients from help-seeking after an episode of self-harm:

“Religion, family, personal problems, and their educational status are major issues which are barriers.” (GP001)

Strong views about suicide were expressed, particularly reflecting the fact that in Islam suicide is prohibited.

“People are usually opinionated about suicide. They say that suicide is a sin according to our religion so people see it as whatever the issue was the person should have taken to solve it through other ways, they shouldn’t have hurt themselves.” (HP005)

### Ideal Care Pathway

#### Talking Treatments

Participants described talking treatments such as counseling, psychoeducation, and problem solving training, which would need to be included if optimal care for patients who self-harm and their family were to be offered:

“There should be counseling services provided for the individual affected or their family. Counseling would prevent them from future acts of self-harm.” (HP006)

Participants suggest that such care should be available in all parts of the health services from primary healthcare units to tertiary care hospitals.

“Problems solving skills should be taught at all the institutes and hospitals.” (HP007)

“In our culture, we do not teach problem solving strategies to adults which is necessary for them because it creates changes in them. If we taught them about problems and also how to solve the problems, they realize their existence in the environment and learn to solve them.” (GP005)

Participants suggested the need for raising awareness of mental well-being strategies at schools, occupational and community settings.

“I suggest awareness programmes at occupational centers and computer centers. There is a need to conduct awareness workshops in schools.” (GP007)

Participants suggest that interventions should be broad and consider both individual and societal risk factors in order to reduce self-harm and optimize mental well-being:

“Psychological work should provide basic psychoeducation. So, people can handle the issues like social or financial. They should be able to know how to make their emotions strong. We should groom our girls as they will move to the next stage; after marriage they will move away. I have seen so many problems in girls regarding how to travel in public spaces, if anyone gets a wrong phone call (from a contact number that they do not recognize) they would be confused, they hesitate, should they pick up the call or not. So, there is a need for education and personal growth so people can make decisions confidently.” (HP004)

There was a difference of opinion about whether religious views on self-harm should be included in any intervention.

“Religiously since harming yourself is a sin, involving the religious aspect of self-harm could result in the patient thinking twice before trying again.” (HP001)

“According to me, religion should not be involved in this training”. (HP004)

#### Clinician Training

All participants emphasized the importance of appropriate training for clinicians about management of mental health problems and self-harm, but reported that they had not received such training:

“No, in Pakistan there is no such trainings as far as medical students are concerned.” (HP006)

Participants suggested that a multi-disciplinary approach to training is needed;

“Being a clinician, it is hard to overcome these problems; we need psychological support as well as psychiatrists as it is a multi-disciplinary approach.” (HP004)

## Discussion

### Summary of Findings

This is the first study exploring self-harm in Pakistan from the perspectives of clinicians. We identified a number of key themes including methods of self-harm seen, perceived motivators to self-harm, own beliefs about people who self-harm, challenges to management, access to care for people who self-harm, ideal pathways, and the need for improved training and supervision of clinicians.

### Comparison With Previous Literature

Participants reported that poisoning was the most common method used by people who self-harm. Previous studies also indicated that medications (benzodiazepines), cleaning products (bleach/bathroom cleaner), rat poison, and organophosphorus compounds (pesticides) are commonly used poisons in self-harm in Pakistan (26, 27).

Participants in this study considered that a variety of factors (including mental health problems, adjustment after marriage, “attention-seeking,” and economic difficulties) were risks factors for self-harm. Clinical guidance for England produced by the National Institute for Health and Care Excellence reviewed evidence from prospective cohort studies and reported depressive symptoms as one of the key risk factor for self-harm and suicide (28). Evidence from Pakistan also support that psychiatric disorders are major predictors of suicide (7). There is well-established evidence for role of low socioeconomic status, low income, and living in poverty as risk factors for self-harm (29–31). Furthermore, adjustment issues after marriage were also highlighted as a potential risk factor. In Pakistan there is huge pressure on women to continue with their unsatisfactory married life which predisposes them to psychological distress and suicidal behavior (32). This however is not unique to Pakistani culture and similar findings are reported in other south Asian countries (33).

The views expressed regarding cultural factors pertaining to Pakistani society include the negative attitudes toward women who self-harm, as they are sometimes viewed not to have a good character. A primary care study from the UK with south Asian women’s views about self-harm and suicide revealed that the concept of *izzat* (i.e., “honor/respect”) is a major influence in Asian families (34). Another study from Pakistan highlighted that the burden of family *izzat* is disproportionately placed upon the women of the family. There are high expectations from women as daughters, daughter in law, sisters, wives and mothers (7). However, the participants in this study also reported that families may physically abuse a male who has self-harmed. Existing evidence also suggests that family members overreact following the self-harm act and express their anger following the attempt (35).

The challenges that clinician reported while managing this group included the legal implications of self-harm. According to Pakistan Penal Code 309 of the Criminal Procedures Act, self-harm and suicide are criminal offenses punishable with a jail term and/or fine (7). More recently, there is some indication that suicidal behavior may be decriminalized in Pakistan. A bill to decriminalize suicide attempt was presented in the Senate in 2017, which was deferred on the grounds that “attempted suicide is a crime” and “forbidden in Islam” (36). Decriminalization would be an important step in eliminating the stigma and addressing the psychological needs of suicidal persons in the country (31).

Hospital-based emergency departments in a low-income country like Pakistan are considered as first point of contact for patients with self-harm and these departments are considered as a window of opportunity for delivering suicide prevention interventions that can potentially life-saving (37). However, lack of human and financial resources in public sector hospitals in Pakistan is a major challenge for quality of care and use of health services (38). Similar views were reported in our current study where clinicians voiced a distinct lack of services as a real challenge while dealing with patients presenting with self-harm.

In our study, stigma, financial constraints and religion were perceived as barriers that prevent people from accessing help after self-harm. Findings from a previous study report that adjectives used to describe people who die by suicide reveals stigmatizing beliefs of themselves as attention-seekers, pathetic, selfish, and weak (39, 40). Refusing to talk about a stigmatized issue is one method of coping with stigma (41); families in Pakistan claim self-harm act as accidental and do not disclose the true nature of the self-harm act because of social stigma (7, 31). There is evidence that poverty and financial constraints are risk factors for self-harm (29–31) and our findings supporting this, clinicians also emphasizing poverty as a potential barrier in accessing help because of lack of resources to pay for treatment costs. Participants reported that religion is a barrier in accessing help. Religious factors influence the diagnosis and registering of self-harm and suicide in Pakistan since in Islam the taking one’s life is forbidden and persons taking their life are considered to be denied entry to heaven (7). Hence people do not report these acts. However, it has been reported that religion may not be as strong a deterrent as was previously believed (31).

Effective management of both medical and psychological factors in emergency departments can have an impact in preventing repetition of self-harm and future suicide (7). Clinicians in this study suggested that access to care was important and that psycho-education should be an essential component for those presenting with self-harm. Previous research conducted with patients, friends and families of people who self-harm reported a number of helpful strategies including: supporting and providing information for carers; family members being included in treatment; and incorporating religious components in treatment (42, 43).

Education and appropriate training for clinicians were also perceived as necessary for the ideal care of people who self-harm. This has been highlighted in previous studies as being associated with more positive attitudes in dealing with patient who self-harm and there is also evidence for improvement in confidence and self-reported attitudes both in general hospitals and in psychiatric staff in Australia and New Zealand (44). A study done in the emergency department of an Irish teaching hospital showed that previous training was associated with increased confidence and greater empathy when managing patients who self-harm (45). There is a need for emergency physicians to be trained in the recognition and management of psychological distress in order to handle self-harm adequately and prevent such attempts in future (7, 46, 47). For medical students in Pakistan, structured internship programmes should be included in their behavioral sciences curriculum.

### Strengths and Limitations

This is the first study to explore clinicians’ (hospital physicians and GPs) views of self-harm in Pakistan, set within a randomized controlled trial of a self-harm intervention. Robust supervision of the research team helped to maintain the credibility and trustworthiness of the data analysis. Our study sample comprised clinicians with a range of clinical experience, which will have impacted on their understanding of and experience in managing people who self-harm.

Interview participants were either physicians working in medical units of public hospitals participating in the CMAP trial, or GPs working in general practices located in the locality of hospitals participating in the trial. Since the majority of the population in Pakistan is served by private sector hospitals, this may mean the experiences and perspectives of physicians in this sector are missing; the views of other healthcare professionals are also missing. Since these views may differ, this is a limitation of the study. It should be considered as an area for future research.

### Implications for Clinical Practice

This study highlights the need for mental health and illness to be recognized as a public health priority, reducing the stigma of mental illness in Pakistan. Lessons could be learned from the “Parity of Esteem” initiative in England: ’Parity of esteem’ is defined as “valuing mental health equally with physical health,” which would result in those with mental health problems benefitting from: equal access to the most effective and safest care and treatment equal efforts to improve the quality of care. This study highlights a clear need for training of *all* doctors, in the identification and management of people with mental health problems, including the management of people who self-harm in order to prevent future self-harm and suicide in Pakistan.

Next steps should include development and evaluation of an intervention, such as specific training courses or clinical treatment protocols, for clinicians who come into contact with adults who self-harm. Such an intervention should be co-produced with input from patients and clinicians, based on research evidence, and its impact and acceptability tested rigorously.

## Conclusions

Understanding clinicians’ views on self-harm, and how these align with broader sociocultural contexts, is necessary for the development and implementation of high quality care, particularly in a culture where self-harm remains an illegal act and is forbidden in their religion. Moreover, it seems that there is need to strengthen clinicians’ capacity by improving their understanding on how to treat patients presenting with self-harm. This will not only help clinicians in more effective management of this target population, but it would also benefit patients, perhaps by reducing the likelihood of repetition of self-harm.

## Data Availability Statement

The data supporting the conclusions of this article will be made available by the authors upon reasonable request.

## Ethics Statement

Ethical approval for the study was granted by the Institutional Review Board of Karachi Medical and Dental College (KMDC) (Ref: 027/15), Karachi, Pakistan, and the ethics committee of the University of Manchester (UoM). The patients/participants provided their written informed consent to participate in this study.

## Author Contributions

NH, NC, CC-G, and PB were involved in designing the study, ongoing supervision and feedback in the process of qualitative analysis, writing the manuscript, and final approval of the manuscript. TK, ST, SF, AQ, CC-G, and PB were involved in development of topic guides, and data analysis. AT reviewed the analysis and drafts of the manuscript. All authors contributed to and approved the final manuscript.

## Conflict of Interest

The authors declare that the research was conducted in the absence of any commercial or financial relationships that could be construed as a potential conflict of interest.
