# Supplementary material for: Clinicians’ Perspectives on Self-Harm in Pakistan: A Qualitative Study
Source: Front Psychiatry. 2021 May 20;12:607549. doi: 10.3389/fpsyt.2021.607549 (PMC8172994; doi:10.3389/fpsyt.2021.607549)
Supplement: Supplementary file 1 [file Data_Sheet_1.docx]

Appendix

**Multicenter RCT to evaluate the clinical and cost-effectiveness of a culturally adapted therapy (CMAP) in patients with a history of self-harm**

**Qualitative research Topic Guide**

**Preamble**

Thank for agreeing to take part in this interview.

Introduce the study and aims of the interview.

Consent

**Doctors/clinicians/GPs**

1: What does the term ‘self-harm’ mean to you?

(prompt-whether all self -harm is life-threatening

(Difference between SH and attempted suicide)

2: Can you describe your experiences of dealing with people who SH?

3: What challenges do you face while dealing with people who present after SH?

(prompt at individual, family, service level)

4: What are the possible causes/reasons that people harm themselves?

Prompt

5. Are there any gender differences:

in reasons for self-harming (prompt : do you think that females might self -harm more for attention seeking, explore reasons for attention seeking)

- presentations (prompt: symptoms, pathways)

-consequences

6. Do you think that women might seek out a female GP?

5: What are the possible consequences of SH?

Prompts – individual (physical/psychological harm), family

Which one is more relevant (if anything) – impact on self or family?

5: In your experience, what are the methods that people usually use in this area to harm themselves?

Prompts

Are there any gender differences

6: What are your observations about family’s attitude towards people who SH?

7: What do you think is the attitude of general public about SH?

Prompts

Are there any gender differences

8: do people seek help before they SH, or after SH?

If so who from?

If not what are the reasons/barriers in accessing help/treatment?

Prompt – Legal/police involvement concerns

What are (or might be) the enablers?

9: What happens to people who SH? Where do they present after SH?

Prompts

Path way

9: What sorts of treatments other than medical/emergency treatment should be available for people after SH? Why? How would they help?

Prompt- explore GPs knowledge/understanding/confidence about psychological intervention

-Role of GPs /Clinicians(current role), and what it should be?

Do GPs provide any kind of counselling/psychological help/psychoeducation?

Prompt : might religion act as a protective factor? Do you ever refer to religion in your interactions with patients?

What do they understand by the term “counselling”?

What type of statements they usually use while educating patients about SH.

Do GPs explore patients’ preference for type of treatment?

10: Where do doctors generally refer people who SH? What might they expect such a referral would achieve? What might the patient expect? Do people tend to attend?

If not, what are the possible reasons?

Prompt

Patients’ and families’ understanding about psychiatrist/psychologist

Preference

11: What sort of interventions should be available for people who SH? Where should these be available/who should facilitate or deliver them? What about training in problem solving skills?

12: What do you understand by the term ‘problem-solving abilities?’

In your opinion is there any relationship/association between SH and problem solving abilities?

If yes, what kind of relationship?

13: What do you think the barriers/enablers for the patients would be? What should we include in that training for the patients?

Prompt – family, religion, personal etc (why?)

14: What is the legal framework for SH and suicide cases?

Prompts:

Process

Is there any need to change?
